# Comparative 2D and 3D Ultrastructural Analyses of Dendritic Spines from CA1 Pyramidal Neurons in the Mouse Hippocampus

**DOI:** 10.3390/ijms22031188

**Published:** 2021-01-26

**Authors:** Maria Nicol Colombo, Greta Maiellano, Sabrina Putignano, Lucrezia Scandella, Maura Francolini

**Affiliations:** Department of Medical Biotechnology and Translational Medicine–Via Vanvitelli 32, Università degli Studi di Milano, 20129 Milan, Italy; marianicol.colombo@unimi.it (M.N.C.); greta.maiellano@studenti.unimi.it (G.M.); sabrina.putignano@studenti.unimi.it (S.P.); lucrezia.scandella@studenti.unimi.it (L.S.)

**Keywords:** excitatory synapse, dendritic spine, electron microscopy, ultrastructure, three-dimensional reconstruction, quantitative analysis

## Abstract

Three-dimensional (3D) reconstruction from electron microscopy (EM) datasets is a widely used tool that has improved our knowledge of synapse ultrastructure and organization in the brain. Rearrangements of synapse structure following maturation and in synaptic plasticity have been broadly described and, in many cases, the defective architecture of the synapse has been associated to functional impairments. It is therefore important, when studying brain connectivity, to map these rearrangements with the highest accuracy possible, considering the affordability of the different EM approaches to provide solid and reliable data about the structure of such a small complex. The aim of this work is to compare quantitative data from two dimensional (2D) and 3D EM of mouse hippocampal CA1 (apical dendrites), to define whether the results from the two approaches are consistent. We examined asymmetric excitatory synapses focusing on post synaptic density and dendritic spine area and volume as well as spine density, and we compared the results obtained with the two methods. The consistency between the 2D and 3D results questions the need—for many applications—of using volumetric datasets (costly and time consuming in terms of both acquisition and analysis), with respect to the more accessible measurements from 2D EM projections.

## 1. Introduction

The concept of the dendritic spine has undergone evolution since their discovery by the neuroanatomist Ramon Y Cajal, in 1888, who observed by means of light microscopy “thin protrusions” on the surface of a number of Golgi-impregnated neurons. These thin structures were further characterized by Gray in 1959 [1], through electron microscopy (EM) investigations, as the major postsynaptic target of excitatory synapses in the central nervous system (CNS). Several decades and numerous morphological and functional studies were needed to finally define dendritic spines as structures that not only increase the synaptic surface but also play an important role in the compartmentalization of biochemical processes within the restricted volume of a spine head [2,3,4]. 

The heterogeneity in size and shape among different brain areas, developmental stages and animal species is a feature of dendritic spines [5,6]. During synaptogenesis, thin filopodia, highly mobile structures protruding from the dendrites, start forming synapses with the axons from nearby neurons [7]. The morphological changes that follow will determine the shape of the spine, if mushroom rather than thin or stubby [8] in a highly mobile and plastic scenario where the final shape of the spine will depend on synaptic activity and strength [9,10,11]. In addition to shapes, also dendritic spine number is highly variable as not only at different ages synaptic establishment and pruning compete but also experience can heavily affect formation of new spines, as well as spine maintenance and elimination [7,12,13]. The overall result of all these developmental and plasticity-related events is the modulation of dendritic spines and synaptic density and ultimately the refinement of synaptic connectivity and neural wiring in the CNS.

Another crucial element to consider when studying excitatory synapses in the CNS is the post-synaptic density (PSD), an electron dense structure, tightly associated with the post synaptic membrane, whose components are essential for neurotransmitter recognition, binding and local downstream signal transduction, and all biochemical processes that trigger plasticity like long term potentiation (LTP). The architecture of the PSD, in terms of size and shape is important to determine both synapse activity and maturation stage. Whereas its thickness, area and the height of the synaptic cleft can vary as a function of activity [14,15,16], the transition from macular to perforated and, sometimes, fragmented PSD has been reported to reflect the shift from immature to mature synapse [2,17].

Because of their functional role in development and in learning and memory processes, changes in dendritic spine morphology, density or maturation are frequently associated to a series of pathological conditions: cognitive impairment, defective motor and learning skills, altered mental status and degenerative diseases, all conditions that are, at least partially, the result of the disruption of neural circuits, due to spine anomalies at the nanoscopic scale or of spine loss [18,19].

Since the size of both the entire spine and the PSD has been tightly associated to the extent of the activity/efficacy of the synapse as a whole [10,13,20], studying dendritic spines size and shapes to correlate these features to specific functional states in physiological and pathological conditions has involved many scientists during the last decades. However the small size and the dense packing of synapses within the neuropil, represent major obstacles for their detailed analysis: conventional bright field microscopy as well as fluorescence confocal microscopy coupled with the use of fluorescent protein constructs first and super resolution approaches later still suffer from resolution limitations, even though they have been and still are invaluable tools for the investigation of spine dynamics both in cultured neurons and in living organisms [21]. Electron microscopy is the best methodological approach for spine structural and especially ultrastructural studies, because of its nanometric level resolution on the X–Y plane [22]. However, even on EM images, synapse recognition and detailed analysis could be challenging, in case the plane of section is not the fortunate one, moreover, in two-dimensional (2D) projection—as any transmission electron microscopy (TEM) image—some important information is lost, such as spine and PSD shape in the third dimension. These issues can be overcome through serial sectioning and imaging with a transmission or a scanning electron microscope (SEM) or serial block face scanning electron microscopy (SBF-SEM), different techniques that allow for the collection of data from a relatively large volume of nervous tissue starting from a series of independent 2D projections. These volumes and the 3D reconstruction of neurons and synapses that can be derived from them represent the pillar of EM-based connectomics [23,24,25,26]. The instruments, protocols and procedures to obtain such datasets are more expensive and labor-intensive [27,28], especially when 3D reconstruction is planned, moreover, depending on the imaging settings, individual 2D projections might be less resolved than those obtained with canonical TEM [22]. While these approaches represent the obvious choice when willing to retrace neuronal connectivity over long distances, they might not represent the best option, or be necessary, when data on individual dendritic spine are needed.

The aim of our work is thus to define if the 3D reconstruction of dendritic spines is needed for the determination of spine features, such as the size and the density by comparing the data obtained from 3D SBF-SEM datasets to the data obtained from 2D TEM projections. We will show here that there is a strong correlation between the results obtained from the analysis of 2D images and the ones that are provided by 3D reconstructed dendritic spines, proving that a less labor-intensive approach, such as measurements on single bidimensional images, is still solid and valuable. However, the preference of 2D analysis should not be considered as a fixed rule: the best strategy must be evaluated case per case, on the basis of various crucial factors, such as the spine parameter we are interested in, the level of accuracy we are searching, the time we can spend for each step of investigation.

## 2. Results

### 2.1. 3D Reconstruction of Dendrites Provides for a Better Picture of Spine Shape with Respect to Transmission Electron Microscopy 2D Projections

**Figure 1:**
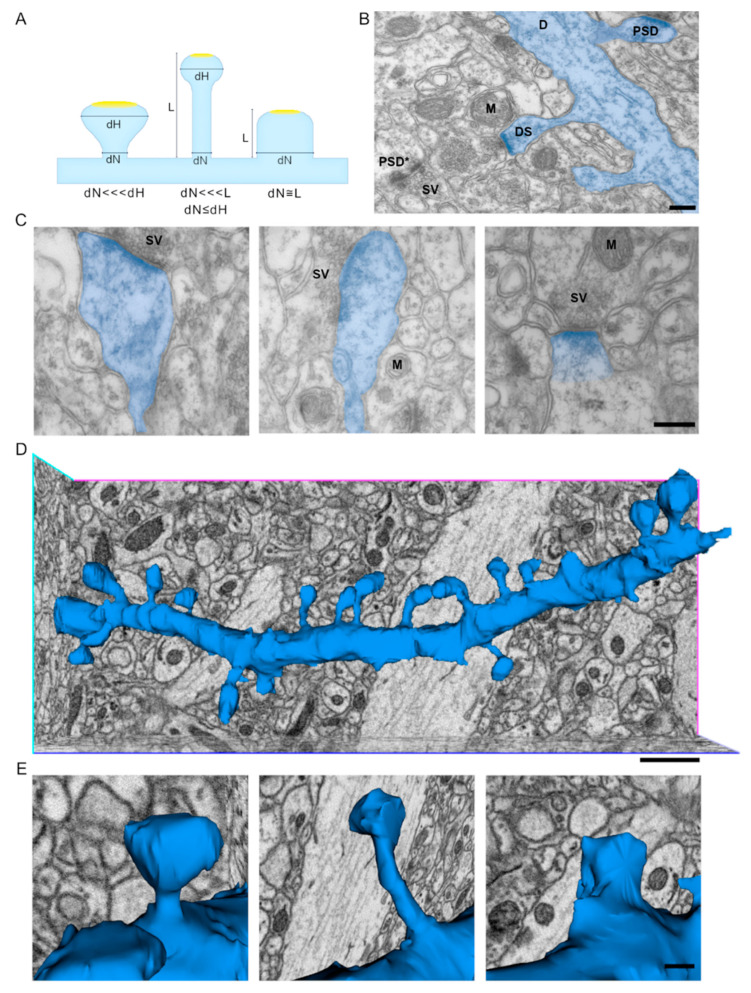
Appearance of dendritic spines in 2D TEM images and in 3D volume rendered dendrite from a volumetric data-set. (**A**) Canonical classification of dendritic spines based on optical microscopy observation and criteria for the attribution of spines to each class, based on the neck (dN) and spine head diameter (dH), and spine length (L). Dendritic spines are defined as mushroom (dN <<< dH), thin (dN <<< L, dN ≤ dH) or stubby (dN≅L); filopodia are not depicted here. Modified from [8]. (**B**) Representative TEM image of mouse hippocampal CA1 neuropil where a segment of a dendrite (D, highlighted in blue) and several excitatory synapses can be seen. In this image it is possible to see few dendritic spines (DS) arising from the dendritic shaft and, within excitatory synapses, the post synaptic densities (PSD) are clearly detectable; among them, a complex post synaptic density (PSD*). The presynaptic terminal is recognizable by the presence of synaptic vesicles (SV). (M: mitochondria; scale bar = 200 nm). (**C**) Representative TEM images of sections of mouse hippocampal CA1 showing respectively mushroom (left), thin (center), and stubby (right) dendritic spines (highlighted in blue) (M: mitochondria, SV: synaptic vesicles; scale bar = 200 nm). (**D**) 3D reconstruction of a representative CA1 dendrite of the mouse hippocampus, the dendrite is segmented and extracted from a stack of independent projections obtained with SBF-SEM (scale bar = 1 μm). (**E**) Higher magnification images of selected spines arising from the dendrite depicted in D. From left to right we can appreciate the 3D aspect of mushroom, thin and stubby spines (scale bar = 200 nm).

For many years now, dendritic spine shape heterogeneity in different brain areas, developmental stages and functional states has been extensively described. Dendritic spines are classified based on their shape thanks to data obtained in optical microscopic studies in which spine shape was highlighted thanks to histochemistry or immunofluorescence/fluorescence approaches and their shape was mostly defined from 2D projections obtained from the optical microscope. The canonical classification of dendritic spines considers the relationship between the diameter and length of the neck and the size of the head. On the basis of these parameters, criteria for the attribution of spines to three different classes have been defined (Figure 1A). Spines are then classified as: (a) mushroom, when the diameter of the head is broader than the diameter of the neck (these characteristics are also shared by cup-shaped dendritic spines); (b) thin, when head and neck diameters are similar, but the neck length is longer than its width; (c) stubby, when the head and neck diameters are similar, and the total length of the spine equals its width. Besides these classes, one further type of long thin protrusions arising from the dendritic shaft has been described, filopodia. The advent of higher resolution fluorescence microscopy approaches, live cell imaging and the possibility to reconstruct in the third-dimension dendrites and spines from independent 2D projections, although allowing for a better comprehension of the shape and dynamics of these important structures, did not dramatically change the way they were classified. In contrast to optical microscopy approaches, the visualization of spines at the ultrastructural level, and thus their classification based on the above-mentioned criteria, can dramatically change depending on the type of sample, electron microscope and protocol of image acquisition used. Indeed, if we compare representative images of dendrites and spines from thin sections of plastic embedded brain tissue examined under a transmission electron microscope, due to the random orientation of the plane of the section and to its limited thickness (roughly 60–80 nm), only occasionally can dendrites be sectioned parallel to their length (Figure 1B), and this is the only situation where it is possible to identify dendritic spines arising from the shaft and unambiguously identify their head and neck, measure their respective width, and the total length of the spine so to properly case them within the classes described above, as mushroom, thin and stubby respectively (Figure 1C). While from these types of 2D projections from single TEM sections it is also almost impossible to decipher which is the morphology of the PSD (simple, perforated, horseshoe or segmented), this type of images offers the possibility to have access to a number of relevant features of the pre-synapse and, here, thanks to the high resolution of the TEM, to investigate this compartment in a qualitative and quantitative manner. Conversely, in the case of a portion of the neuropil reconstructed from 3D datasets (that can be obtained through serial sectioning of the embedded samples, or through backscattered scanning electron microscope imaging of the resin block face, such as FIB (focused ion beam) or SBF (serial block face) SEM), as reported here, having access to the entirety of a dendrite (Figure 1D), the classification of the spines arising from its shaft could be done in the most precise manner as, in most cases, the head and neck can be clearly identified and individual spines can be categorized as mushroom, thin or stubby, as shown here (Figure 1E). These spines are amenable to different quantitative measurements (volume of the spine head, size of the PSDs, architecture of the PSD etc.), some of which will be considered here. A further advantage from using 3D reconstruction of dendrites is that by looking at each individual spine, per each single dendrite, the frequency of spine shape per dendrite can be evaluated, and this might be of interest when following spine changes that are associated with maturation during development or in pathological conditions.

### 2.2. Stereology as a Favourable Approach for Spine and Synapse Density

**Figure 2:**
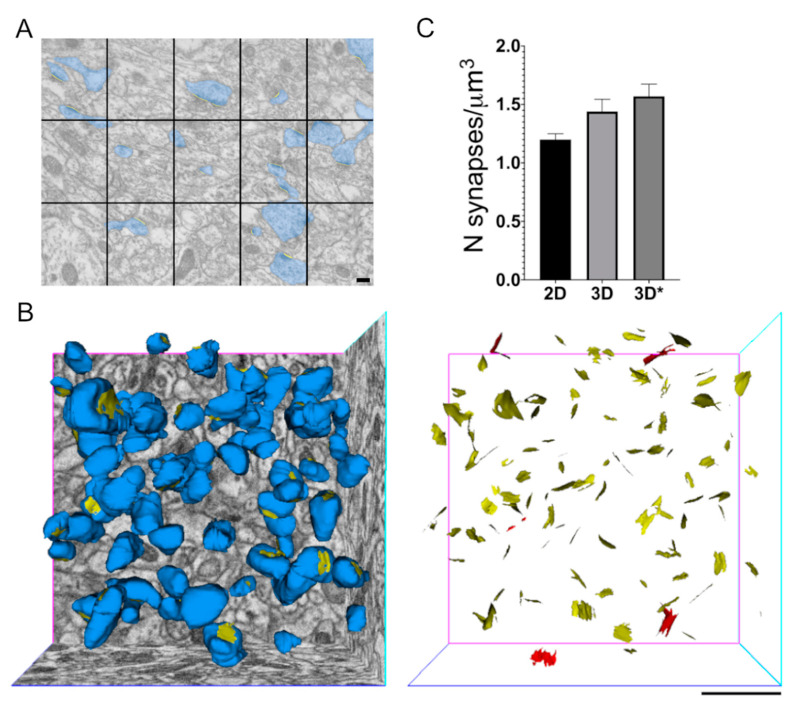
Effects of the type of sample, imaging and strategy adopted on synapse density evaluation. (**A**) Representative TEM micrograph of the mouse CA1 hippocampus where a number of excitatory synapses (highlighted in blue) can be easy detected and counted by the superimposition of a stereological grid, according to the size-frequency method (see Materials and Methods). The PSDs counted are highlighted in yellow (scale bar = 200 nm). (**B**) Reconstructed spine heads (blue) and SASs (yellow) in a volume of CA1 hippocampal neuropil (left); and isolated SASs on dendritic spines (yellow) and SASs on the dendritic shaft (red) (right) (scale bar = 1 μm). (**C**) Evaluation of excitatory synapse density comparing the results obtained with stereology on 2D EM projections and count on 3D reconstructed volumes. The histogram reports the mean number of synapses/μm^3^ ± standard error of the mean (S.E.M.) for each type of analysis and both count of only dendritic spine SASs (3D) and these, together with the dendritic shaft SASs (3D*) are reported.

When the density of dendritic spines and synapses in the neuropil are investigated, the practical problem of having a single thin section to evaluate the density of any specific feature in a 3D tissue/organ is once again the main limitation. However, in past years, different stereological approaches have been established to evaluate excitatory synapse density per μm^3^ starting from counts on 2D sections, but this data will always result from an approximation. To compare the results from a stereological approach and the manual count on 3D reconstructed volumes, we analysed a total neuropil surface of roughly 1900 μm^2^, from the apical dendrite region of mouse CA1 hippocampus, and evaluated excitatory synapse density from 75 TEM micrographs acquired at 19,000× and 25,000× magnification. Here, the synaptic density has been estimated by means of the superimposition of stereological grids on TEM images (Figure 2A) and synapses were counted and their density evaluated using the size-frequency method (see Material and Methods). On the other side, in the reconstructed neuropil of the same brain area, we counted the number of synapses in a volume of 190 μm^3^. Synapses either only on spine heads (Figure 2B, left panel) or these together with synapses on dendritic shafts (Figure 2B, right panel) have been counted, and in both cases, synapses intersecting the three excluding-planes have not been taken in consideration. The excitatory synapse mean density (Figure 2C) resulting from the stereological analysis is comparable (2D), even if slightly lower, to those obtained from the 3D analyses (3D). The mean value of density measures obtained by counting PSDs within volumes is higher in the third column (3D*), due to the inclusion in the count of synapses on the dendritic shaft. There are no significant statistical differences among these results indicating that, first, stereological approaches still represent a strong and reliable method to evaluate synapse density from TEM bi-dimensional images, and second, that in the brain area under exam here, the number of excitatory synapses on dendritic shaft is much smaller than the number of synapses on spine heads and its contribution to the total amount of excitatory asymmetric synapses is very limited. For the quantitative evaluation of synapses reported in Figure 2C, the tissues processed for TEM and analysed with stereology derived from three different mice whereas the 3D data were obtained from different blocks of tissue (processed for SBF-SEM) from two of those three mice.

### 2.3. Spine Features Related to Their Function Can Be Evaluated with Both 2D and 3D EM Datasets

**Figure 3:**
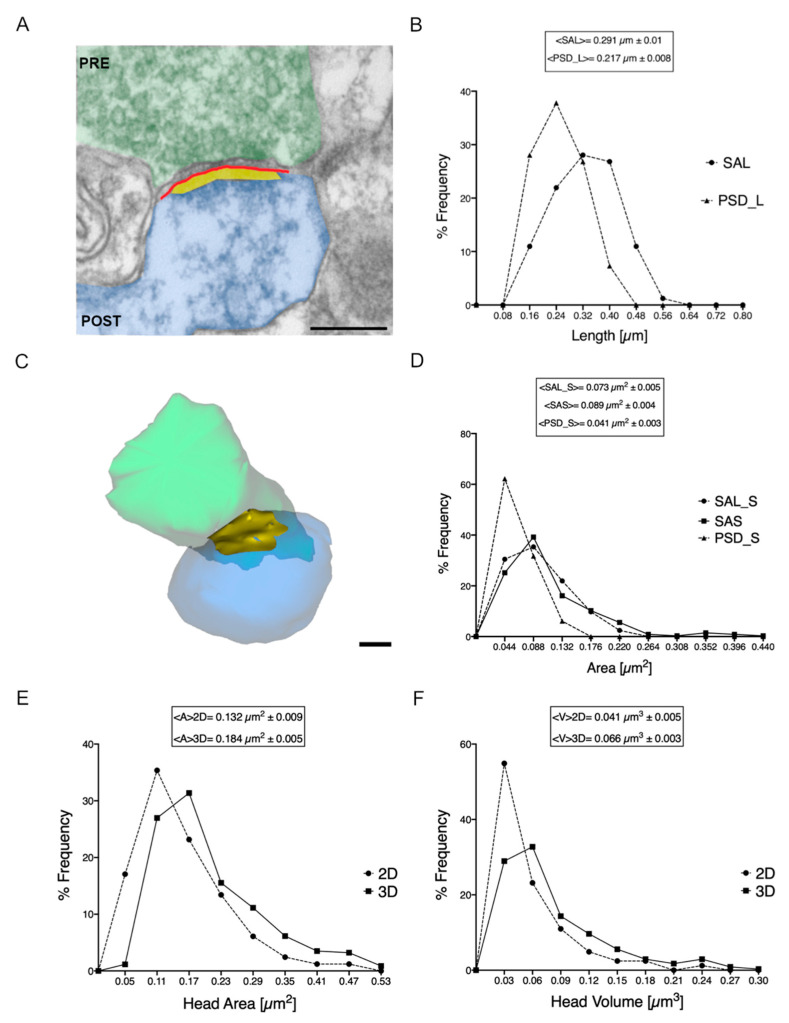
Comparative analyses of spine head area and volume, SAS and SAL between the measures obtained on 2D and 3D data-sets. (**A**) Representative excitatory synapses from 2D TEM image. The pre- and post-synaptic compartments are highlighted in green and blue respectively. The yellow region represents the PSD, whereas the red line represents the synaptic apposition length (SAL), see text. (scale bar =200 nm); (**B**) Distribution curves of the SAL (dashed line-circles; <SAL> = 0.291 μm ± 0.010) and PSD length (dashed line-triangles; <PSD_L> = 0.217 μm ± 0.008) measured and calculated in 2D TEM micrographs; (**C**) Representative excitatory synapses from 3D reconstruction. The pre- and post-synaptic compartments are highlighted in green and blue respectively. The synaptic apposition surface (SAS) can be visualized, in yellow, as a shell shaped structure (scale bar=100 nm); (**D**) Distribution curves of the SAS (continuous line-squares; <SAS> = 0.089 μm^2^ ± 0.004), SAL_S (dashed line-circles; <SAL_S> = 0.073 μm^2^ ± 0.005) and PSD_S (dashed line-triangles; <PSD_S> = 0.041 μm^2^ ± 0.003) are presented; (**E**,**F**) Distribution curves of areas and volumes of the spine heads measured and extracted in 2D and 3D as detailed in Materials and Methods. The curves have been generated through the partitioning of spine heads area and volume into 9 and 10 classes of frequency, respectively. The curve of the area measured in 2D (dashed line; <A> 2D= 0.132 μm^2^ ± 0.009) and the one calculated from 3D volumes (continuous line; <A> 3D = 0.184 μm^2^ ± 0.005) are reported in (**E**). Similarly, for spine head volume, the distribution curve of volumes calculated from 2D (dashed line; <V> 2D = 0.041 μm^3^ ± 0.005) and the one of volumes measured in 3D (continuous line; <V> 3D = 0.066 μm^3^ ± 0.003) are reported in (**F**). All data are reported as the mean value ± S.E.M.; these values derive from the analysis of more than 80 dendritic spines from 2D TEM sections and more than 340 spines segmented and reconstructed into 3D objects from samples of mouse CA1 hippocampus.

**Figure 4:**
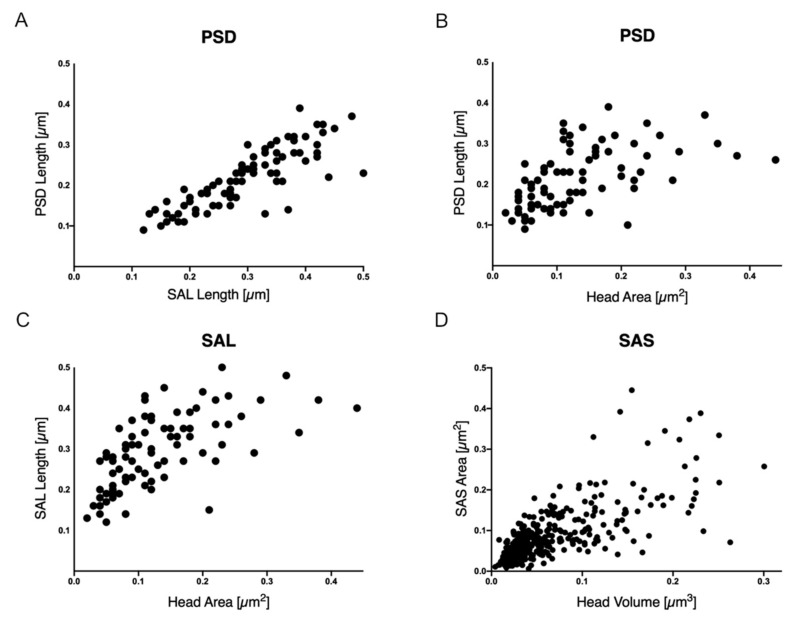
Relationship between the spine head area and volumes with PSD length, SAL and SAS. The correlation between these parameters is reported as graphic representation here: (**A**) SAL length (μm) and PSD length (μm); (**B**) spine head area (μm^2^) and PSD length (μm); (**C**) spine head area (μm^2^) and SAL length (μm); (**D**) spine head volume (μm^3^) and SAS area (μm^2^). These values derive from the analysis of more than 80 dendritic spines from 2D TEM sections and more than 340 spines segmented and reconstructed into 3D objects from samples of mouse CA1.

The correlation between spine head and PSD size with synaptic function is an accepted fact [10,20], underlying the relevance of measuring these parameters when studying synapses at the ultrastructural level. We then next investigated if these variables can be faithfully evaluated by measuring either on 2D sections with a TEM or on 3D datasets. For TEM sections, asymmetric synapses complying with the criteria indicated on the Materials and Methods were considered. For each sectioned spine, we measured the area of the head, the length of the PSD (PSD_L) and, in addition, we evaluated the length of the juxtaposition between the pre- and postsynaptic membranes (SAL) also defined as axon-spine interface (ASI) on volumes [29] (Figure 3A). We considered this further aspect of the head of the spine as it was recently suggested that, in neurons of rat hippocampal organotypic slices [30], the dimension of the head might not always reflect the size of its PSD and vice-versa. We then compared the 2D measurements of the length of the PSD and SAL obtained from TEM sections of spines. Looking at the distribution of the lengths, the SAL ranged from 0.120 to 0.505 μm, with a mean value of 0.291 μm ± 0.010 (S.E.M.), while PSDs were slightly shorter (as expected, considering their relation with SAL, as depicted in Figure 3A) measuring from 0.089 to 0.385 with a mean length of 0.217 μm ± 0.008 (S.E.M.). The frequency distribution curves, obtained by dividing the distribution length of the SAL and the PSD in 10 bins (intervals of 0.08 μm; Figure 3B), were slightly different in shape even if they present a significant overlap and, as expected, the PSDs are shorter than the juxtaposition of the pre- and post-synaptic membranes. Interestingly, the two values were tightly correlated (Figure 4A).

From the 3D-rendered reconstructed spines, we have calculated the volume of the head and the synaptic apposition surface (SAS), that is the surface of apposition between the PSD at the post-synaptic membrane and the presynaptic membrane where the active zones (AZ) are present [31,32] (Figure 3C). To make a comparison between the measure obtained from 2D and the 3D data we had approximated the area of the head (from 2D data) to a circle and the volume of the head (from 3D data) to a sphere. By means of mathematical approach (see Material and Methods) we converted the volume measured in 3D into an area (Figure 3E) and the area obtained in 2D analyses into a volume. We set the area of the head as the widest section of a sphere (Figure 3F).

Spine head area ranged from 0.021 to 0.439 μm^2^ for 2D measures, with a mean value of 0.132 μm^2^ ± 0.009 (S.E.M.), while the area extrapolated from 3D measures ranged from 0.031 μm^2^ to 0.542 μm^2^, with a mean value of 0.184 μm^2^ ± 0.005 (S.E.M.). We draw a distribution frequency curve (Figure 3E), by dividing the area values in 9 bins of 0.06 μm^2^, and even if the two curves (2D and 3D) are not completely overlapping, they follow the same trend, showing a peak of frequency in correspondence of the smallest values 0.05–0.17 μm^2^. The values of areas measured in 2D suffered from underestimation linked to the plan of sectioning, therefore values of areas from 3D appear higher. Spine head volumes extrapolated from 2D measures, ranged from 0.002 to 0.219 μm^3^ with a mean value of 0.041 μm^3^ ± 0.005 (S.E.M.), while 3D measurements indicated a minimum value at 0.004 μm^3^ and a maximum at 0.300 μm^3^, with a mean value of 0.066 μm^3^ ± 0.003 (S.E.M.). Looking at the volume frequency distribution curves (Figure 3F) obtained dividing the volume range in 10 bins (0.03 μm^3^ intervals), once again, despite the origin of the data plotted here, both curves showed a similar progression with a peak at small volumes (more than 75% of spine heads had a volume lower than 0.1 μm^3^), with only few spines whose volume was broader than 0.12 μm^3^. Strikingly when both area and volume of spines were considered, measures obtained on 2D projections tended to be slightly smaller, both as mean value and as size frequency distribution. This observation suggests that even if the data collected are statistically different (*p* < 0.0001), because the numerical values of area and volume measured in 2D may suffer from a certain underestimation possibly linked to the intrinsic limitations of measuring in 2D a 3D object and of the plan of sectioning, the overall message that is conveyed by 2D measures is comparable with that of 3D measures.

Considering the SAL, its surface, calculated from 2D measures, varied from 0.011 to 0.200 μm^2^, with a mean value of 0.073 μm^2^ ± 0.005 (S.E.M.) while SAS evaluated from 3D datasets, measured from 0.006 to 0.445 μm^2^, with a mean value of 0.089 μm^2^ ± 0.004 (S.E.M.). These data strongly correlated when the frequency distribution curves of SAL and SAS (Figure 3D) were examined, the curves almost fully overlapped following the same trend and sharing similar average values. When we plotted the values of the surface of the PSD derived from 2D measures (from 0.006 to 0.116 μm^2^, with a mean value of 0.041 μm^2^ ± 0.003) on the frequency distribution curve, it greatly overlapped with SAL and SAS curves. The statistical analyses of the data collected do not show any difference between the SAL_S and SAS measurements, while a strong variance is present between these data and the PSD_S (*p* < 0.0001). Indeed, once again and not surprisingly, the surface of the PSD is reduced with respect to both SAL and SAS surfaces.

We then measured the correlation between the length of the PSD and the SAL and the length of PSD, the SAL and the SAS with the area or the volume of the head of the spine respectively. The data, analysed with the Spearman’s rank correlation coefficient, showed a good correlation between the parameter analysed: SAL and PSD length (r = 0.85) (Figure 4A), the spine head area and the PSD length (r = 0.62) (Figure 4B), the spine head area and the SAL (r = 0.70) (Figure 4C), and spine head volume and the SAS (r = 0.73) (Figure 4D).

## 3. Discussion

The aim of our work is to evaluate whether analyses on single-section TEM micrographs and reconstruction from 3D-EM stacks are equally reliable and give comparable results, when dendritic spines from asymmetric excitatory synapses in the central nervous system are qualitatively and quantitatively described.

For many decades, transmission electron microscopy studies, where individual thin sections of resin embedded brain samples were examined at high resolution, have been the only method to gain insight into the ultrastructure of the neuropil, neurons, neurites and synapses [33] (and references therein). However, despite the huge amount of details and information that were obtained from the analyses of these bi-dimensional projections, soon the lack of information from the third dimension emerged and a number of subsequent pioneer studies addressed this absence of data through very elegant experiments in which hippocampal synapses and spines were reconstructed thanks to the acquisition of stacks of independent images from TEM serial sections [34] (and references therein) [8,35]. It is only in the last two decades that, thanks to the advent of scanning electron microscope (SEM) imaging of the tissue block face by means of the backscattering contrast, and to the possibility to sequentially section the block (like in the serial block face-SEM or SBF-SEM), or to mill out the surface of the block itself (like in focused ion beam-SEM or FIB-SEM) in the SEM chamber, huge amount of three-dimensional ultrastructural data could be obtained, allowing for the segmentation and reconstruction of big volumes of neuropil [36,37,38] (and references therein). Finally, from a combination of the two approaches and thanks to an improved and automated sectioning procedure, a new method to obtain z-stacks examined under a SEM for the 3D segmentation and reconstruction of broad volume of neuropil has been established and it is represented by the automated tape-collecting ultramicrotomy with scanning-EM (ATUM-SEM) [37,39] (and references therein). Altogether these methodological approaches led to the possibility of obtaining information of the neuropil ultrastructure on the x, y and z axes, with different spatial resolution, from large volumes of tissue, and this wealth of information has been functional to study neuronal connections over long distances and ultrastructural connectomics. In ultrastructural connectome studies, two methodological aspects are of great relevance: the minimal required resolution and the volume (related to the minimal dimension of the neural circuits that are the object of the study) [40,41] (and references therein). All technical approaches used in these studies must offer the best compromise between these two requirements and, in such scenario, SBF-SEM and ATUM-SEM are the two methods that allow for the best match between resolution and volume. Both methods are indeed used for connectome studies [28,42] (and references therein) but the advantage of SBF-SEM with respect to systems based on serial sectioning is the fact that with the former no complex and time-consuming alignment and distortion corrections are required as images in the data-set are inherently aligned due to the unique method of 2D projection generation and acquisition [36,37]. On the other hand, these issue alignments can heavily affect the processing of serial sectioning-based data-sets as occasional loss of sections, folding and the presence of wrinkles might represent a great challenge. Conversely, all approaches based on SEM imaging of serial sections have the great advantage of being conservative, allowing to repeatedly examine the same sections [37].

These improved 3D techniques are becoming increasingly popular in the scientific community, so much that one might question if there is still room for analyses of individual neurons and synapses based on TEM studies and importantly, if all observations and data that were and will possibly be obtained by means of TEM analyses still hold validity and if the results obtained by such an approach are comparable and compatible with the results obtained by 3D analyses.

To answer this question, we started by considering the classification of dendritic spines as traditionally defined from optical microscopy studies. Spines can be morphologically divided into different varieties, based on the shape and relative dimension of their neck and head, as mushroom and cup-shaped, thin, stubby and filopodia. We have shown that with both the approaches (analyses from 2D and 3D datasets) it is possible to identify and classify dendritic spines however, when using 2D TEM images from thin sections, the orientation of the plane of the section and its thickness represent an important limitation, allowing the proper recognition of the head and neck of the spine only occasionally. On the contrary by using volume reconstructed dendrites and spines, spine shape can be easily defined and spines classified. The limitation in the use of a 2D dataset is important if the architecture of the PSD is investigated (i.e., simple, perforated, horseshoe, fragmented). Indeed, even if the initial morphological characterization of this structure was done with TEM [43,44], the distinction among these different types of PSD cannot be fully appreciated on a bi-dimensional projection regardless to the plane of the section. Undoubtedly, if this aspect has to be addressed the use of a 3D reconstruction of the spine head becomes the preferential form of analysis [45]. Conversely, the high resolution 2D TEM micrographs of spine heads are extremely useful to measure PSD length and thickness [46,47,48], and curvature [16].

The second aspect we considered here, is the asymmetric excitatory synapse density that we measured on TEM sections with stereology and by counting spine heads and PSDs in 3D reconstructed volumes. Interestingly the two independent approaches yield comparable results and is consistent with what we have previously reported in [48], where the use of both methods enabled us to evaluate and compare asymmetric synapse density in the hippocampus of mice with two different genotypes. Hence the use of TEM bi-dimensional images in the study of spine density in different experimental conditions (such as animal species, brain areas, developmental stages, genotypes or disease models) leads to a great saving of time without affecting the quality of the result. When we considered the contribution of PSDs on dendritic shafts to the overall number of PSDs in the hippocampal volume examined, we found that they represent only a small fraction of the total and this is in agreement with previous reports highlighting the uneven distribution of asymmetric synapses on spines and on shafts in the mouse cortex [6,40].

The scenario is more complex when we considered quantitative parameters of the dendritic spine head like area and volume. The results obtained from 2D and 3D measures (processed as described above to allow for comparison), varied between the two datasets when the average values were considered, but these differences were mostly abolished when we looked at the data considering their frequency distribution curves. These observations suggest that the 2D measures underestimated the real spine head dimensions [49], which are more faithfully recorded in reconstructed volumes. The differences, when considering the mean values, could be explained by the fact that, in order to compare data from 2D and 3D datasets, we approximate the head of the spine to a sphere and thus we introduced in our measures an element of simplification that can partially account for the underestimation of the head dimensions in 2D. Moreover, even if we carefully identified the spines to measure on our TEM images (according to the parameters that were described in Materials and Methods), we could never be sure that the section of the spine head we considered corresponded to its maximum diameter/surface, thus the reported area could be slightly underrated. It has to be stressed here, however, that when these measures on 2D images are used to compare synapse features in different experimental configurations, the same bias equally affects all analyses thus it has no effect on the reliability of the comparison.

In our study we found similar values, both in terms of mean and frequency distribution, between the SAS and the SAL, when evaluated with 2D and 3D approaches, while comprehensibly, the PSD was shorter with respect to the SAL and reduced in extension with respect to the SAS. Indeed, the SAS do not precisely correspond to the PSD area, as it represents the surface of apposition between the active zone on the presynaptic terminal and the PSD on the post synaptic terminal measured on volumetric datasets [32]. Although this discrepancy could partially be attributed once again to sectioning for TEM imaging, in our case this is mostly due to the poorer lateral resolution in the 3D stacks, compared to the higher resolution 2D micrographs. The lateral resolution of the projections within the stacks did not always allow to precisely define the PSD limits and led to an overestimation of SAS areas, when compared with the dimensions measured form the 2D TEM images. Thus, this overestimation of the SAS is possibly due to the inclusion in the measure of a portion of the postsynaptic membrane that encircles the actual PSD. These considerations tell us that the lateral resolution is crucial in the study of synapses as in most cases differences between experimental datasets can be tiny (i.e., the PSD length and thickness) and the resolution/sensibility of the acquisition system has to match and to be able to detect these differences. It is to note that when the highest spatial resolution (in both 2D and 3D) is required to study specific features of synapses and spines, TEM is by far the most powerful instrument when tomography [50] and/or cryo-tomography [51,52] (and references therein) are used. Indeed, thanks to these applications, nanometer and sub-nanometer resolution can be obtained. In most cases, however, the increase in resolution is accompanied by a reduction in the volume that can be reconstructed [53] and, to date, most TEM tomography and whole-cell cryo-tomography studies on synapses report data obtained from in vitro cultured neurons; this is mainly due to the intrinsic limitations of cryo-immobilization and cryo-TEM sample preparation procedures. To partially overcome the limitations in the thickness of the sections observed under a TEM for tomography applications and thus the limitations in the accessible volume, the use of high voltage TEM allowing the acquisition of tilt series from sections up to 1–2 μm thick has been proposed. Moreover, tilt series from serial sections can be acquired and, after accurate alignment, these stacks can be used to 3D reconstruct larger volumes of brain tissue [54], or synapses and neurons grown in vitro [55].

Our analyses finally showed that there is a correlation between the size of the SAS and the volume of the head of the spine even if it is not as tight as those reported in previous studies [8,34,49] and this correlation is also present when we correlate the SAL and PSD length together and both of them with the area of the head. A further support of our claim about the solidity of the 2D measure of the PSDs to morphologically compare dendritic spines in different experimental paradigms, is represented by our previous results demonstrating that PSD analyses of length from 2D TEM images and volumes from SBF-SEM data-sets were equally able to highlight differences in the morphology of this structure in hippocampal neurons from brains of wild type mice with respect to those of a mouse model of X-linked intellectual disability [47].

To conclude, the choice between the 2D or the 3D approaches to describe in a qualitative and quantitative manner dendritic spines in defined area of the brain neuropil, must be carefully evaluated on the basis of the main parameters of interest and its variability interval and the type of analysis needed. It is also always important to remember that in most cases, a compromise between spatial resolution and volumes has to be reached thus, a combined use of the two methods can be appropriate. Finally, an important aspect that has to be considered is the possibility to have access to the appropriate devices for image acquisition and image processing, that in the case of volume-EM datasets can be an important limitation. Once ascertained that the reconstruction of the neuropil in the 3D model represents a favorable approach for spine and PSD shape definition, it remains to verify if the manipulation of high amount of data generated by the 3D approaches can be considered indispensable for the study of the features of interest in dendritic spines.

## 4. Materials and Methods

In this study, different samples for transmission electron microscopy (TEM) and serial block face scanning electron microscopy (SBF-SEM) were used. These mouse brain samples have previously been processed and used for other purposes and the results published in prior studies. All the experimental procedures followed the guidelines established by the Italian Council on Animal Care and were approved by the Istituto Superiore di Sanità (ISS) and Italian Ministry of Health (approval numbers 27/2010, 747/2015-PR, 275/2015 and 322/2018) as reported in previous publications [47,48,56]. All efforts were made to minimize the number of subjects used and their suffering. Thin sections for TEM and SBF-SEM volumetric data-sets were consistently obtained from regions of the *stratum radiatum* parallel to the proximal apical dendritic field at a distance of 120–150 μm from the soma of hippocampal CA1 pyramidal neurons of adult (P60-P90) C57/Bl6 mice whose brains were processed as described earlier [47,48,56]. Briefly, after intracardial perfusion of deeply anaesthetized mice (by intraperitoneal injection of 4–5% chloral hydrate) with paraformaldehyde (2–4%) + glutaraldehyde (1–2.5%) containing fixatives, brains were dissected and coronal vibratome (Leica VT 1000S, Leica Microsystems, Wetzlar, Germany) sections (100–200 μm) were obtained. From these sections, the CA1 region of the hippocampus was manually isolated and the trimmed samples were further processed for EM. For TEM, samples were postfixed with osmium tetroxide (2%) and en-bloc stained with a saturated solution of uranyl acetate dissolved in 20% ethanol. After complete dehydration, samples were embedded in Epon-Spurr epoxy resin that was baked at 60 °C for 48 h. Thin sections (60–70 nm) were obtained with the ultramicrotome (Leica UC 4/6, Leica Microsystems, Wetzlar, DE) by means of a diamond knife (Ultra 45°, Diatome Ltd., Nidau, Switzerland) and collected on 300 mesh copper grids. Sections were further on-grid stained with a saturated solution of uranyl acetate dissolved in bidistilled water and 1% lead citrate.

For SBF-SEM, trimmed samples were treated with reduced 2% osmium solution containing 1.5% potassium ferrocyanide and then treated with 1% thiocarbohydrazide followed by a further incubation in 2% osmium tetroxide. Samples were en-bloc stained with 1% uranyl acetate (aqueous) and with Walton’s lead aspartate solution. After dehydration, samples were embedded in Durcupan or Epon hard epoxy resins that were cured at 60 °C for 48 h. Unless otherwise indicated, all reagents were purchased from Sigma-Aldrich (St. Louis, MO, USA) and Electron Microscopy Sciences (Hatfield, PA, USA). For the two-dimensional (2D) TEM analyses reported here, images were collected using a CM10 transmission electron microscope operated at 80 kV (FEI, Eindhoven, Netherlands) with a Morada CCD camera (Olympus, Munster, Germany) at final magnification of 19, 25, 34 and 46.000 ×. For three-dimensional (3D) SBF-SEM analyses, data-sets were acquired in a Gatan 3View2XP (Gatan Inc. Pleasanton, CA, USA) mounted on a Zeiss Sigma VP SEM (Carl-Zeiss, Oberkochen, Germany) at 2.0–2.5 KV in high and low (10 Pa) vacuum mode, with pixel size ranging from 6 to 25 nm in the x, y axis and 50 nm in the z axis.

### 4.1. Quantitative Morphometric Analysis of Dendritic Spines from 2D-TEM Micrographs

We considered in our study only asymmetric synapses in which the PSD and the synaptic cleft were clearly visible and distinguishable and at least three synaptic vesicles could be clearly identified in the presynaptic compartment. For each spine section we measured the spine head area, the post-synaptic density (PSD) length (PSD_L) and the synaptic apposition length (SAL), defined as the length of juxtaposition between the membranes of the synaptic bouton and the spine. Synaptic density on 2D EM projections was calculated using the size-frequency method [57], where the estimate of the number of synapses/µm^3^ (*Na*) can be obtained by using the Equation (1):

*Na* = (*Nd*/*A*)/*d*, (1)

with *Nd* represents the number of synaptic profiles that are not touching the edges of a stereological grid, *A* is the examined surface (µm²), and *d* is the average length of PSDs (µm). Thus, we generated stereological grids that were superimposed onto EM images (75 images randomly acquired at final magnification of 19.000–25.000×), for a total of 1900 µm^2^ surface analysed. The analysis was performed on non-adjacent portions of the neuropil that did not contain axons, broad primary dendrites or cell bodies. All analyses of TEM micrographs at different magnifications were done with Fiji 1.53c.

### 4.2. Reconstruction of Dendrites and Spine Heads from 3D Data-sets

The 3D reconstruction of dendrites and spines was performed using the software Espina 2.8.2 [58]. To make our comparison between quantitative parameters of dendritic spines obtained from 2D and 3D EM, we manually segmented and reconstructed all spine heads that were included in given volumes of roughly 300 μm^3^, excluding those that intersected the edges of the frame. On these reconstructed spines, we measured the volume of the head and the area of the synaptic apposition surface (SAS), as previously described by Morales and co-workers [32]. The segmentation of the SAS is done automatically by the software upon the manually identification of the asymmetric synapse. The density of excitatory synapses was calculated by the manual count of spine heads and PSDs, an approach that has proven to be the most sensitive [59]. We reconstructed and analysed roughly 200 μm^3^ of neuropil (from different data-sets/mice) and we excluded from the counted synapses those which intersected the three excluding edges of each sub volume.

### 4.3. Strategies to Compare the Data from 2D and 3D Objects

In order to ease the comparison of spine head measures obtained from 2D micrographs and 3D reconstructions, we approximate the shape of the head to a sphere [32] and the shape of the SAS to a circle. After the valuation of the radius of the sphere, using Equation (2)

R = ∛((V/π) × 3/4),(2)

where r = radius and V= volume of a sphere, we calculate the area of the circle A (Equation (3))

A = πr^2^, (3)

that we assumed is comparable to the area of the section we measured in 2D micrographs. 

The other way around, for the shift from 2D measurements to 3D reconstruction data, we estimate that the area of the dendritic head that we measured in 2D micrographs can be considered as the largest section of a sphere and after finding the radius (inverse of Equation (3)), we obtained the volume (inverse of Equation (2)). Then we calculate the theorical 2D SAS considering the PSD_L and the SAL as its the diameter, using Equation (4)

A = π × (2r/2)^2^. (4)

### 4.4. Data Analysis

We used an Excel Spreadsheet to collect data, calculate the derived data, mean, standard errors and standard deviation. The graphs were generated with GraphPad Prism 9. Statistical analyses were performed with GraphPad Prism 9 using either the Mann–Whitney test or the one-way ANOVA test for single or multiple comparison, respectively. The Spearman correlation test was conducted to analyse the correlation between two different parameters.

## Data Availability

The data presented in this study are available on request from the corresponding author.
